# A Challenge for Engineering Biomimetic Microvascular Models: How do we Incorporate the Physiology?

**DOI:** 10.3389/fbioe.2022.912073

**Published:** 2022-06-20

**Authors:** Arinola O. Lampejo, Nien-Wen Hu, Daniela Lucas, Banks M. Lomel, Christian M. Nguyen, Carmen C. Dominguez, Bing Ren, Yong Huang, Walter L. Murfee

**Affiliations:** ^1^ J. Crayton Pruitt Family Department of Biomedical Engineering, University of Florida, Gainesville, FL, United States; ^2^ Department of Mechanical and Aerospace Engineering, University of Florida, Gainesville, FL, United States

**Keywords:** angiogenesis, lymphangiogenesis, microcirculation, tissue engeneering, biomimetic

## Abstract

The gap between *in vitro* and *in vivo* assays has inspired biomimetic model development. Tissue engineered models that attempt to mimic the complexity of microvascular networks have emerged as tools for investigating cell-cell and cell-environment interactions that may be not easily viewed *in vivo*. A key challenge in model development, however, is determining how to recreate the multi-cell/system functional complexity of a real network environment that integrates endothelial cells, smooth muscle cells, vascular pericytes, lymphatics, nerves, fluid flow, extracellular matrix, and inflammatory cells. The objective of this mini-review is to overview the recent evolution of popular biomimetic modeling approaches for investigating microvascular dynamics. A specific focus will highlight the engineering design requirements needed to match physiological function and the potential for top-down tissue culture methods that maintain complexity. Overall, examples of physiological validation, basic science discoveries, and therapeutic evaluation studies will emphasize the value of tissue culture models and biomimetic model development approaches that fill the gap between *in vitro* and *in vivo* assays and guide how vascular biologists and physiologists might think about the microcirculation.

## Motivation

Almost every tissue in our bodies has blood and lymphatic vessels. Growth and remodeling of these vessels involves multiple cell types and can be associated with most diseases. Consequently, designing therapies to combat pathological conditions spanning tumor metastasis, diabetic retinopathy, islet transplantation, skin graft survival, and tissue ischemia necessitates understanding the cell dynamics involved in microvascular remodeling and how cells interact with each other in response to microenvironmental molecular cues. A challenge, however, is observing cells over the time course of these processes. The most common time-lapse *in vivo* approaches include multi-photon microscopy in the brain (Berthiaume et al., 2018; Bonney et al., 2021), dorsal window chamber preparations (Peirce et al., 2004), and the use of zebrafish (Gore et al., 2012; Venero Galanternik et al., 2017; Gore et al., 2018). The need for higher throughput and tunable methods has motivated an emerging area of research focused on biomimetic microvascular model development.

At the intersection of tissue engineering and physiology, biomimetic microvascular model development overlaps with lab-on-a-chip and organoid design with the end goal being to recapitulate the complexity of a real, microvascular network environment. Most common *in vitro* models can be characterized as bottom-up approaches that add one, two, or three cell types into a matrix environment (Kaunus et al., 2011). More technologically influenced approaches involve patterning cell and/or matrix through, for example, bioprinting or microfluidic based platforms (Song and Munn, 2011; Wang et al., 2016; Haase and Kamm, 2017; Campisi et al., 2018; Osaki et al., 2018; Henderson et al., 2021; Shirure et al., 2021; Ren et al., 2022). In recent years, these models have enabled reductionist experiments focused on isolating the effects of the individual components. A limitation of these systems is that they do not look like real microvascular networks and as a result engineers struggle to convince physiologists of their approach’s relevance.

The objective of this mini-review is to overview the recent evolution of popular biomimetic modeling approaches for investigating microvascular dynamics and highlight the potential of the mesentery tissue culture model to fill the gap between *in vitro* and *in vivo* models. By emphasizing key players involved in microvascular remodeling, we identify key characteristics that can be considered model design requirements. We then provide examples of how our work in recent years has established the mesentery tissue culture model and its use for making scientific discoveries. Recognizing that every model has limitations and that a model is only as valuable as the scientific questions it is used to answer, our overview and examples frame the future potential for biomimetic models.

## Key Players in Microvascular Remodeling

**FIGURE 1:**
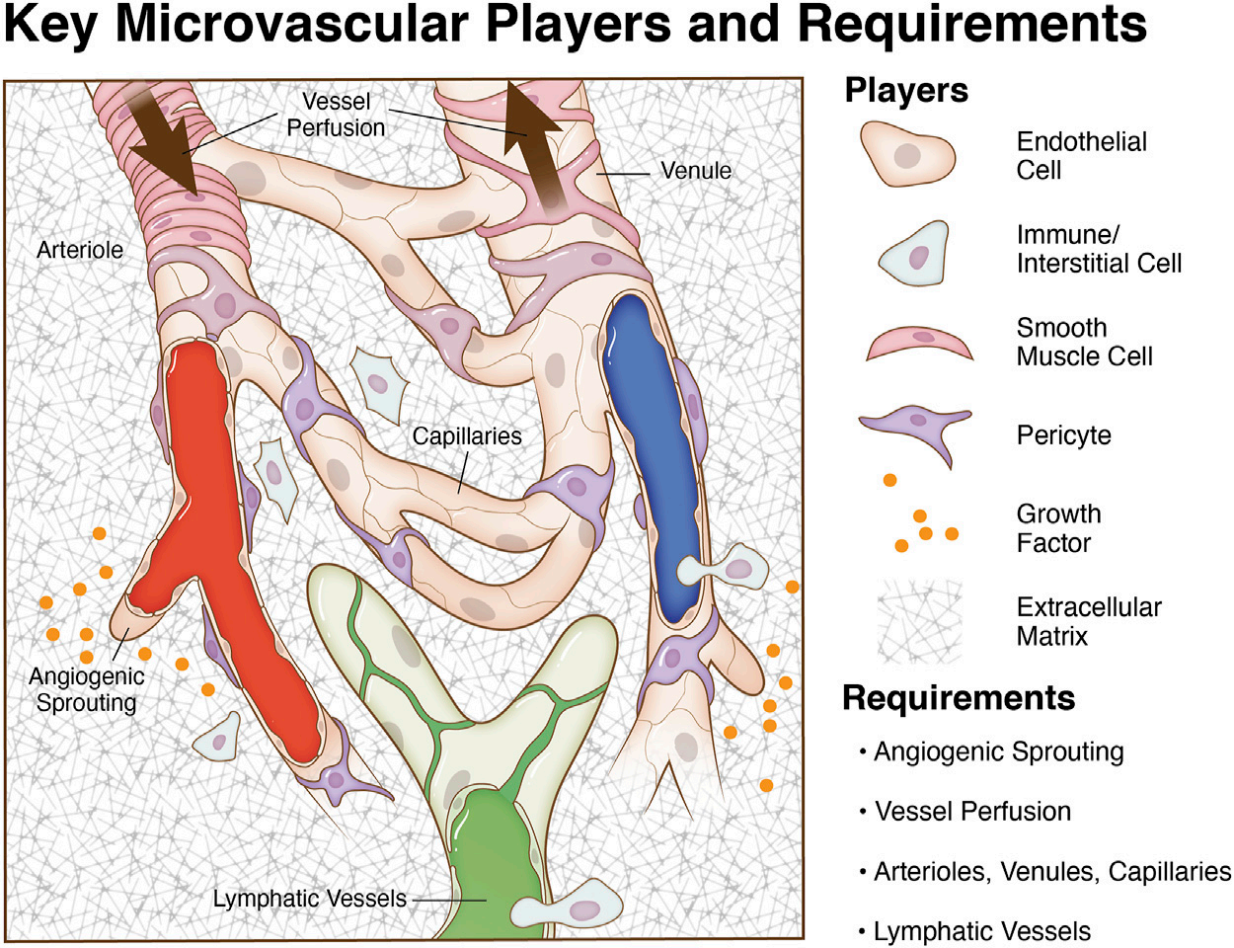
Key microvascular players and model requirements. The microcirculation consists of blood vessels and initial lymphatic vessels. Mimicking the complexity of an intact microvascular network during angiogenesis and lymphangiogesis entails the incorporation of multiple cell types in system relevant patterns, chemical cues, vessel perfusion, and an appropriate matrix environment. A goal for tissue engineering and biomimetic microvascular model development is to recapitulate the multi-cell/system interactions and vessel functionality.

One of the main advantages that can help biomimetic models provide a more physiologically relevant environment in which to study new microvascular dynamics is the incorporation of multiple cell types. Endothelial cells, pericytes, smooth muscle cells, and macrophages all play key functional roles in a network microenvironment. Processes such as angiogenesis, vasculogenesis, and arteriogenesis, rely on the interplay between these different cell types. Thus, understanding the specific functions that endothelial cells, pericytes, smooth muscle cells, and macrophages have on their environment and on each other can help discover new phenomena that could be critical to more accurately modeling different disease pathologies. More importantly for the context of this article, incorporation of these players and characteristics can be viewed as design requirements for developing a biomimetic microvascular model (Figure 1).

### Endothelial Cells

Endothelial cells are key regulators of multiple important vascular mechanisms. Endothelial cells control the uptake of various molecules and proteins into the circulatory system, sense and respond to changes in shear stress, modulate vessel dilation and constriction, and regulate inflammatory processes. One of the most important functions that endothelial cells participate in is angiogenesis, characterized by the growth of new blood vessels from existing vessel networks. During angiogenesis, nearby endothelial cell adhesions are weakened, which allows for the formation of a capillary sprout from an existing vessel. Capillary sprouts are comprised of a migrating tip cells and proliferating stalk cells. Following paracrine signals released from supporting perivascular cells, capillary sprouts migrate from the vessel of origin into the extracellular matrix and can eventually form connections with other vessels or capillary sprouts. A more extensive review of endothelial cell functionality can be found here (Carmeliet, 2000; Gerhardt and Betsholtz, 2003; Peirce and Skalak, 2003; Kelly-Goss et al., 2014).

### Pericytes

Another cell type that participates in remodeling and angiogenesis is the pericyte. Pericytes, which are characterized by markers such as smooth muscle actin, PDGFR-β, and neuron-glial antigen 2 (NG2), act as support cells along capillaries. Evidence suggests they play an important role in regulating vessel diameter, influencing vascular permeability, stabilizing vessels through direct and paracrine interactions, and promoting endothelial cell survival and proliferation. During angiogenesis, pericytes work with other mural cells to deposit components of the basement membrane such as laminin and collagen which support the growth of new capillary sprouts. Pericytes can also help guide and stimulate outgrowth of sprouts by secreting VEGF, bFGF, and other important growth factors. Additional references on the role of pericyte in angiogenesis are provided here (Murfee et al., 2006; Kelly-Goss et al., 2014; Stapor et al., 2014).

### Smooth Muscle Cells

Smooth muscle cells are mural cells that reside along larger arterioles and venules. Smooth muscle cells regulate several vascular dynamics including maintenance of vessel function, constriction, and dilation. While mostly associated with vasoregulation (i.e., diameter control) and characterized by the expression of contractile proteins, smooth muscle cells can also play a critical role in the initiation of angiogenesis. For example, smooth muscle cells help start the process by detaching from blood vessels in response to Ang2 stimulation. This results in an increase in vascular permeability which triggers vasodilation and destabilization of endothelial adhesions. For more comprehensive discussion of the affect of smooth muscle cell on vascular dynamics see the references (Lilly, 2014; Motherwell et al., 2017).

### Macrophages

Finally, macrophages are another cell type that warrant inclusion due to their presence in inflammatory environments. Macrophages can be sorted into two separate subgroups based on their role in inflammation. M1 macrophages are pro-inflammatory macrophages that can secrete cytotoxic agents while M2 macrophages more commonly secrete anti-inflammatory agents that stimulate angiogenesis. In addition to paracrine mechanisms, macrophage involvement in angiogenesis has also been suggested to include guiding capillary sprouting *via* local extracellular matrix degradation, facilitating pruning and maturation of vessel segments, and even transdifferentiating into endothelial cells (Corliss et al., 2016; Du Cheyne et al., 2020). Macrophages also play an important role in remodeling in disease settings, as shown by their roles as pro-angiogenic cells in the tumor microenvironment (Fu et al., 2020) as well as their upregulation of MMPs during pathological processes including tumor growth, HIV, and multiple sclerosis (Bar-Or et al., 2003; Webster and Crowe, 2006). The relative contribution of the various mechanisms during angiogenesis remains to be comprehensively evaluated and additional evidence for phenotypic overlaps with pericytes and observations of pericyte-like behavior continues to be uncovered, suggesting that our understanding of macrophage involvement in angiogenesis is incomplete. See these references for more detailed review of macrophages interacting with the microvasculature (Corliss et al., 2016; Du Cheyne et al., 2020).

While a model’s necessary level of complexity and whether a model can satisfy the requirements for physiological relevance remain to be debated, it is important to emphasize that developing a microvascular model depends on the incorporation of specific cell types. Each cell type can be freshly isolated from tissues or obtained commercially. Critical decisions include consideration of cell origin (e.g., microvessels versus macrovessels or arterial versus venous) and phenotypic drift during culture. In order to mimic the microvasculature, multiple cells must be spatially assembled in relevant patterns. As an alternative bottom-up approach to build vessels, stem cell populations have been used as heterogeneous cell sources based on the premise that stem cells can undergo appropriate differentiation or even self-assemble into capillary networks (Kusuma et al., 2013; Zanotelli et al., 2016; Jones et al., 2021; Tracy et al., 2022). Regardless of the approach, the tissue engineering challenge of mimicking physiological relevance is highlighted by comparison to a real network (Figure 1).

Now consider the coordination between angiogenesis and lymphangiogenesis, the analogous growth of initial lymphatic vessels, the influence of local growth factor and matrix cues, the potential involvement of other cell populations, and the importance of local hemodynamics. Altogether multiple cell types and systems dance in concert to make up the architecture of a perfused microvascular network. For example, lymphatic and blood vessel coordination important for tissue homeostasis and the presence of lymphatic vessels has been shown to influence angiogenesis—an effect thought to be related to competitive binding of common growth factors (Sweat et al., 2012). Lymphatic vessels also are able to transdifferentiate into blood vessels (Azimi et al., 2020a). Other cell populations influencing capillary sprouting include fibroblasts, which can secrete growth factors and cytokines, and/or interstitial precursor cell populations, which can also secrete factors and differentiate into vascular cells. As for local hemodynamics, shear stress has been linked to angiogenesis as it is a regulator of endothelial cell behavior and phenotypes (Song and Munn 2011; dela Paz, et al., 2012; Galie, et al., 2014; Baeyens, et al., 2015; Driessen, et al., 2018; Peacock, et al., 2020). Recognizing the importance of multi-cellular/system dynamics, critical questions remain regarding temporal relationships, cell plasticity, and cell-cell interactions. To complicate issues, answers to these questions depend on the environment and what milieu of players are present. From a vascular biologist’s point of view, physiological relevance can be qualified by the multi-cellular/system complexity of a functional network.

## Overview of *In Vitro* Biomimetic Microvascular Model Approaches

In microvascular research, *in vitro* models provide a platform to study various growth and remodeling dynamics that may not be discernable *in vivo*. Common *in vitro* approaches used to study angiogenesis and lymphangiogenesis include three-dimensional (3D) cell culture models, bioprinting, and microfluidic devices.

### Cell Culture Models

Basic cell culture systems incorporating endothelial cells, fibroblasts, or a combination of the two or more cell types cultured on basement membrane or Matrigel matrix have been used to study the formation of networks *via* anatamosing cords (Emonard et al., 1987; Vernon et al., 1992; Donovan et al., 2001). 3D cultures aimed at studying angiogenesis can usually also incorporate co-culture of different cell types but are distinguished from 2D systems by their incorporation of tunable gels or biomaterials that better mimic extra cellular matrix. 3D models have thus excelled in allowing researchers to study vacuolation, lumen formation, and integrin-dependent matrix remodeling during network formation, angiogenesis, and vasculogenesis (Bayless et al., 2000; Stratman et al., 2010; Krishnan et al., 2008; Darland and D’Amore, 2001; Stratman et al., 2009). Cutting-edge 3D models characteristically involve culturing endothelial cells and support cells to create networks of multi-cellular vessels. The ability to tune the matrix composition, include multiple cell types, and introduce interstitial flow allows for investigating vessel assembly and capillary network formation.

### Bioprinting

Bioprinting represents an approach for controlling the spatial patterning of specific extracellular matrix proteins and/or cell types. More recently, the use of bioprinting technologies has emerged to create vessels within thick 3D matrix structures. In one example that highlights the state of the field, sacrificial inks have been printed in thick tissue constructs and then removed afterwards to create perfusable channels (Ren et al., 2022). Such perfusable channels are connected to a perfusion system, mimicking large vessels to supply nutrients and oxygen to and remove wastes from nearby tissues and secondary vascular structures.

### Microfluidic Models

Two main microfluidic platforms dominate the literature. In one platform, perfused endothelial cell lined channels are separated by a matrix region (Song and Munn, 2011; Campisi et al., 2018). Endothelial cells are able to elongate and migrate into the matrix region and connect with vessels originating from the other side. Importantly the new network of endothelial cell segments becomes perfused. The seeding of cells, for example cancer cells, into the matrix region enables investigation of cell trafficking to the vessels. Physiological relevance is increased within this platform by tuning the material used for patterning the channels and the verification of vessel permeability (Qiu et al., 2018). Finally, an application for investigating lymphangiogenesis is made possible by seeding the channels with lymphatic endothelial cells (Osaki et al., 2018; Henderson et al., 2021). The second common microfluidic platform for mimicking the microcirculation is best described by a matrix region with an inlet and outlet channel (Wang et al., 2016; Shirure et al., 2021). Seeding the matrix region with endothelial cells and mural support cells resulted in the assembly of a perfusable capillary network with connections to both the inlet and outlet channels.

Each modeling strategy has advantages and disadvantages and depending on your scientific question or objective the required complexity could vary. 3D cell culture experiments have provided useful information on how different matrices and spatial cues affect vascular function (Lee et al., 2014; Knezevic et al., 2017; Robering et al., 2018), but often lack flow or feature limited interactions with other microvascular cell types. While microfluidic models are beneficial because of their ability to incorporate flow (Barkefors et al., 2009; Kim et al., 2016; Yamamoto et al., 2019), these models still face drawbacks such as a limited number of incorporated cell types and a lack of physiologically relevant microvascular network interactions. And with regards to bioprinting, assembly of vessels with multiple cell layers and small vessels with a diameter less than 50 µm remains challenging. All models have limitations, and the impact of the limitations depends on whether they impact the interpretations of results. Models with fewer players allow focusing on specific interactions, yet the questions remain—how complicated does a biomimetic model need to be and does a model with fewer players adequately mimic a real tissue? Regardless of the model, verification of physiological relevance is paramount.

## Filling the Gap With the Mesentery Culture Model

**FIGURE 2:**
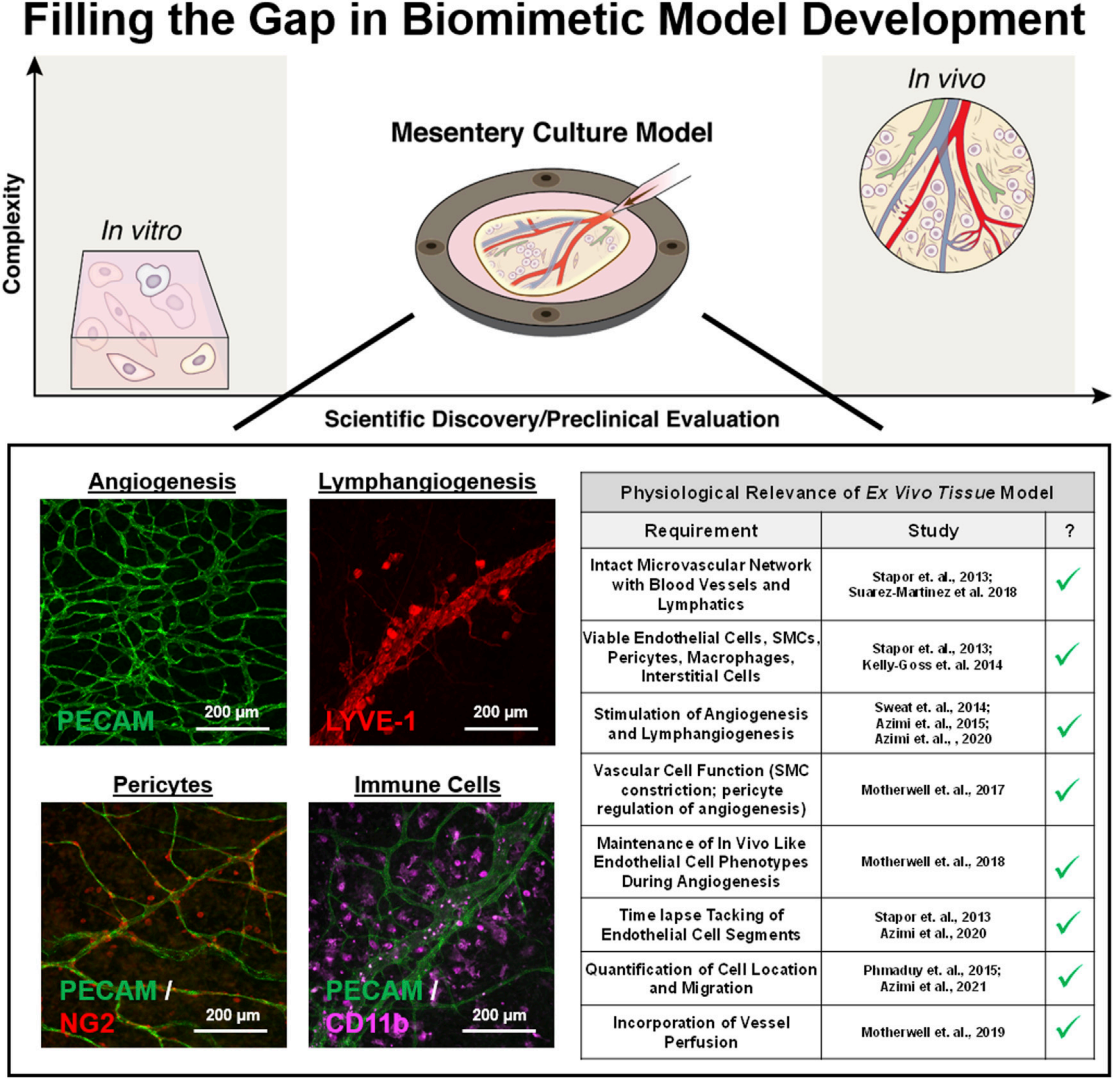
Filling the gap in biomimetic microvascular model development. The mesentery culture model aims to fill the gap between *in vitro* and *in vivo.* Recent work has demonstrated the utility of the approach for investigating angiogenesis and lymphangiogenesis at the same time. Multi-cellular complexity is highlighted by the presence of intact networks, endothelial cells, smooth muscle cells, pericytes, and immune cells. Cells display expected vessel-specific morphologies, and the model’s physiological relevance is supported by maintained vasoreactivity, *in vivo* like cell phenotypes, and the incorporation of vessel perfusion.

Compared to the *in vitro* approaches, tissue culture models represent a top-down approach to recreating *in vivo* complexity by maintaining players *in situ*. Tissue culture models are not new; for example, using brain slice tissue explants has been a common tool for acute physiological studies (Stoppini et al., 1991). The aortic ring assay, in which slices of aorta are cultured in a 3D gel and multi-cellular sprouts grow radially outward over the time course of days, is probably the most common tissue culture model used to study angiogenesis (Nicosia and Ottinetti, 1990; Nicosia et al., 1994). Advantages include the maintenance of endothelial cell and pericyte dynamics while a weakness is the unknown relevance of investigating sprouting from a larger macro-vessel structure. An analogous tissue culture model is the lymphatic ring model, which can be used to visualize lymphangiogenic sprouting dynamics (Bruyère et al., 2008; Iqbal et al., 2017; Katakia et al., 2020). Both ring models do not incorporate flow and cannot be used to study angiogenesis and lymphangiogenesis at the same time. Whether both models include microvascular networks is debatable. Conceivably for the aortic ring model, the new radial vessel segments could originate from the aortic endothelial cell layer or the adventitia’s vasa vasorum. Also, the radial segments can connect to each other. Regardless, the resulting radial segments do not mimic the hierarchy of vessels associated with a microvascular network composed of arterioles, capillaries, and venules. An alternative approach is to use tissues with intact microvascular networks such as retina explants (Murakami et al., 2006). Because of the ease of harvesting and historical use for intravital studies, our laboratory has recently focused on using rat and murine mesenteric tissues and has developed a mesentery culture model (Figure 2). This culture model enables time-lapse investigation of cell—cell interactions at specific locations across blood and lymphatic microvascular networks.

### Model Introduction

The mesentery culture model can be used with tissues from both rats and mice, although the use of mouse tissues requires pre-vascularization of the tissues before harvesting. Mesenteric windows, defined as the translucent connective tissues found between artery/vein pairs feeding the small intestine, are harvested starting from the ileum section. The model is simple and entails cutting tissues out and culturing them (Stapor et al., 2013; Azimi et al., 2017) post harvesting, in individual wells in a 6-well culture plate. During culture, minimum essential media can be supplemented with serum or specific growth factors to stimulate network growth. The mesentery tissue is attractive for the development of a biomimetic microvascular model that mimics the complexity of a real *in vivo* microenvironment. Mesentery tissue is 20–40 μm thick, making it ideal for culturing and imaging. The cell dynamics involved in angiogenesis in the mesentery reflect those in other tissues and it has been used for the evaluation of microvascular function. Rationales for using mesentery beyond its simple culture protocol include the tissue’s historical use to gain foundational insights about lymphatic physiology, cell dynamics during angiogenesis, and microvascular function.

### Physiological Verification

During culture, blood endothelial cells, pericytes, smooth muscle cells, interstitial immune cells, lymphatic endothelial cells, and even nerves can remain viable (Stapor et al., 2013; Sweat et al., 2014; Suarez-Martinez et al., 2018a; Hodges et al., 2020). The physiological relevance is further supported by the demonstration of the functional effects of pericytes on endothelial cell sprouting, smooth muscle cell contraction along arterioles, the maintenance of *in vivo* like endothelial cell phenotypes along with capillary sprouts during angiogenesis, and preferential vessel sprouting of capillaries and venules versus arterioles (Figure 2; Stapor et al., 2013; Motherwell et al., 2017; Motherwell et al., 2018; Azimi et al., 2020b). We have also demonstrated the ability to induce lymphangiogenesis (Sweat et al., 2014) and network perfusion which can introduce physiologically relevant flow velocities in capillaries during culture (Motherwell et al., 2019). Single-pass perfusion was accomplished using a peristaltic pump in series with the biochamber placed inside an incubator set to standard culture conditions. Flow passed through the vasculature and drained out of the venous side to be collected in a waste reservoir. A major difference highlighting the trade-offs between biomimetic approaches is that compared to microfluidic systems, which have controlled inlet and outlet ports (Song and Munn, 2011; Akbari et al., 2017; Hasse and Kamm, 2017; Shirure et al., 2021), the open loop system in the mesentery model allows control of fluid velocities, but not pressures—a difference that highlights the trade-offs between different biomimetic approaches.

Importantly, physiological verification of the mesentery culture model motivates future experiments. Published data supports tissue viability out to 7 days (Stapor et al., 2013) when cultured in serum free media and unpublished data suggests that tissues can remain viable out to 2 weeks. However, in serum free media smooth muscle and pericyte coverage become less consistent by 5–7 days (Stapor et al., 2013) and networks start to lose their hierarchical organization. Vessel perfusion has only been maintained out to 2 days and the impact of perfusion on hierarchical cell and vessel structure remains to be determined. Another important characteristic to evaluate is possible phenotypic drift at later time points. Motherwell et al. (2018), demonstrated that serum stimulated capillary sprouts after 3 days of culture display similar VEGF-R2, UNC-5b, and CD36 expression patterns compared to angiogenic sprouts *in vivo* and an earlier study confirmed that NG2-positive pericytes remain functional during angiogenesis in culture over the same time duration (Stapor et al., 2013). While these results support maintenance of phenotypes, additional studies focused on other cell types are needed.

### Impact on Discovery

The mesentery tissue culture model’s potential impact on scientific discovery is highlighted by the ability to view cell dynamics over the time course of network remodeling and to deliver unique comprehensive readouts including vessel permeability, endothelial cell junctional integrity, lymphatic/blood vessel malformations, cancer cell migration, and cancer cell invasion. Recently, we utilized double transgenic lineage mice to discover the ability of pericytes to detach from vessels (Payne et al., 2021) and migrate into the interstitial space during angiogenesis. We also have observed endothelial cells jumping off of one capillary sprout and connecting with another neighboring sprout (Suarez-Martiniez et al., 2018b). The observation of endothelial jumping resonates with the discovery of vascular island incorporation as a new type of endothelial cell dynamic during angiogenesis. Time-lapse imaging of disconnected endothelial segments confirmed their ability to connect with nearby networks during remodeling (Kelly-Goss et al., 2012; Kelly-Goss et al., 2013; Stapor et al., 2013). The value of our model is maybe best supported by the time-lapse visualization of vessel malformations associated with lymphatic-to-blood vessel transition—a discovery also made possible by the unique view of the model (Azimi et al., 2020a). Evidence for lymphatic-to-blood vessel transition in cultured tissues is supported by 1) real-time tracking of lymphatic segments, 2) observation of the formation of lymphatic-to-blood and blood-to-lymphatic vessel connections, and 3) loss of both LYVE-1 and podoplanin labeling along remodeling initial lymphatic vessels (Azimi et al., 2020b). The ability to watch where cells go and how cells interact with one another during microvascular remodeling will undoubtedly offer new insights by combining the approach using mouse and rat tissues with bioprinting and cell transfection technologies to enable tracking of endogenous and exogenous cell types.

### Potential for Future Applications

The physiological relevance and ability to track cell populations uniquely position the mesentery tissue culture to make a big impact, especially through more applied studies focused on evaluating microvascular interactions with exogenous cells. For example, recent studies have demonstrated the feasibility of anti-angiogenic drug testing and adding various stem and cancer cell populations into the mesentery microenvironment (Azimi et al., 2015; Burks et al., 2016; Azimi et al., 2020a; Suarez-Martinez et al., 2021). These advances prime the utility of the model to investigate drug testing and the therapeutic effects of cell targeted therapeutic strategies during angiogenesis and lymphangiogenesis. In addition, the use of real tissues enables the evaluation of these dynamics within aging or disease settings (Hodges et al., 2018).

## Conclusion and Challenges for the Field

Mimicking *in vivo* complexity is a key challenge for biomimetic model development and highlights a disconnect between tissue engineers and physiologists or vascular biologists. Regardless of the approach (bottom-up or top-down), key characteristics will not be included. Such a reality provokes a more appropriate question: What is the value of a biomimetic model? Future discussion is warranted, yet an important note remains—any model is only as good as the question being asked. So moving forward, we suggest that a more appropriate question for connecting research perspectives should be related to the discoveries and applications made possible by innovative approaches versus traditional *in vivo* studies.
